# New Horizons in Hyperpolarized ^13^C MRI

**DOI:** 10.1007/s11307-023-01888-5

**Published:** 2023-12-26

**Authors:** Myriam M. Chaumeil, James A. Bankson, Kevin M. Brindle, Shdema Epstein, Ferdia A. Gallagher, Martin Grashei, Caroline Guglielmetti, Joshua D. Kaggie, Kayvan R. Keshari, Stephan Knecht, Christoffer Laustsen, Andreas B. Schmidt, Daniel Vigneron, Yi-Fen Yen, Franz Schilling

**Affiliations:** 1 Department of Physical Therapy and Rehabilitation Science, University of California, San Francisco, CA USA; 2 Department of Radiology and Biomedical Imaging, University of California, San Francisco, CA USA; 3 Department of Imaging Physics, The University of Texas MD Anderson Cancer Center, Houston, TX USA; 4 Cancer Research UK Cambridge Institute, University of Cambridge, Cambridge, UK; 5 Department of Biochemistry, University of Cambridge, Cambridge, UK; 6 NVision Imaging Technologies GmbH, 89081 Ulm, Germany; 7 Department of Radiology, Addenbrooke’s Hospital, University of Cambridge, Cambridge, UK; 8 Cancer Research UK Cambridge Centre, Cambridge, UK; 9 Department of Nuclear Medicine, TUM School of Medicine, Klinikum Rechts Der Isar, Technical University of Munich, Munich, Germany; 10 Department of Radiology, Memorial Sloan Kettering Cancer Center, New York City, NY USA; 11 Molecular Pharmacology Program, Memorial Sloan Kettering Cancer Center, New York City, NY USA; 12 Weill Cornell Graduate School, New York City, NY USA; 13 The MR Research Centre, Department of Clinical Medicine, Aarhus University, Palle Juul-Jensens Boulevard 99, Aarhus, Denmark; 14 Partner Site Freiburg and German Cancer Research Center (DKFZ), German Cancer Consortium (DKTK), Im Neuenheimer Feld 280, 69120 Heidelberg, Germany; 15 Division of Medical Physics, Department of Diagnostic and Interventional Radiology, Medical Center, Faculty of Medicine, University of Freiburg, Killianstr. 5a, 79106 Freiburg, Germany; 16 Department of Chemistry, Integrative Biosciences (Ibio), Karmanos Cancer Institute (KCI), Wayne State University, Detroit, MI 48202 USA; 17 Athinoula A. Martinos Center for Biomedical Imaging, Department of Radiology, Massachusetts General Hospital, Harvard Medical School, Boston, MA USA

**Keywords:** Consensus, Hyperpolarized 13C, Metabolism, Metabolic imaging, MR spectroscopy, MRI

## Abstract

Hyperpolarization techniques significantly enhance the sensitivity of magnetic resonance (MR) and thus present fascinating new directions for research and applications with *in vivo* MR imaging and spectroscopy (MRI/S). Hyperpolarized ^13^C MRI/S, in particular, enables real-time non-invasive assessment of metabolic processes and holds great promise for a diverse range of clinical applications spanning fields like oncology, neurology, and cardiology, with a potential for improving early diagnosis of disease, patient stratification, and therapy response assessment. Despite its potential, technical challenges remain for achieving clinical translation. This paper provides an overview of the discussions that took place at the international workshop “New Horizons in Hyperpolarized ^13^C MRI,” in March 2023 at the Bavarian Academy of Sciences and Humanities, Munich, Germany. The workshop covered new developments, as well as future directions, in topics including polarization techniques (particularly focusing on parahydrogen-based methods), novel probes, considerations related to data acquisition and analysis, and emerging clinical applications in oncology and other fields.

## Introduction

Hyperpolarization techniques can increase the inherently low sensitivity of magnetic resonance (MR) by more than four orders of magnitude [1]. They have opened fascinating new avenues for *in vivo* MR imaging and spectroscopy (MRI/S). Hyperpolarized ^13^C MRI/S allows real-time metabolic changes to be observed non-invasively in living organisms, including cells, tissues, animal models, and humans. In clinical research studies, hyperpolarized ^13^C metabolic imaging has shown the potential to non-invasively improve diagnosis and monitoring of therapy in patients without the use of ionizing radiation. The only hyperpolarization technique applied in human research studies, dissolution dynamic nuclear polarization (d-DNP), was introduced 20 years ago and demonstrated the ability to detect [1-^13^C]pyruvate metabolism in animals 15 + years ago [2], representing a breakthrough in MR metabolic imaging. However, there are many challenges for hyperpolarized ^13^C MR to be translated to a routine clinical tool, from overcoming technical challenges (polarization, probes, and acquisition) to identifying the best clinical applications [3]. To accomplish the overwhelming promise of hyperpolarized ^13^C MR to revolutionize medical diagnostic imaging, efforts are ongoing to improve the technique, investigate new applications, and find the best use cases for the technology. Evaluating the added clinical value of hyperpolarization techniques is currently a major challenge with more than twenty ongoing clinical trials focusing on hyperpolarized [1-^13^C]pyruvate, the poster child of metabolic probes that employ the technique [4–6]. Novel technologies are being developed to lower the hurdle for clinical translation and allow a more widespread and reliable use of the technique. During an international workshop on “New Horizons in Hyperpolarized ^13^C MRI” which took place on the 13th of March 2023 in the Bavarian Academy of Sciences and Humanities, Munich, Germany, recent developments ranging from hyperpolarization technology, novel probes, acquisition strategies, competing/synergistic technologies, and clinical studies were presented and discussed. In this article, we review the developments that were presented and summarize the scientific exchange that occurred during the workshop.

## Latest Advances in Hyperpolarized ^13^C MR

### Technical Progress of d-DNP

The basics of d-DNP and its recent applications have been thoroughly described in a recent textbook [7]. Recent advances in this field have mostly been on the clinical side, and are discussed here and in “Clinical Studies: Scope, Acquisition, and Analysis.” A major milestone in clinical application of hyperpolarized ^13^C MRI came with the SPINlab d-DNP polarizer [8], enabling human studies at over 24 sites. This system processes up to four samples simultaneously and includes automated quality assessment steps. Recent advances in d-DNP technology include the advent of cryogen free polarizers [9, 10], polarization at high field [11], faster polarization using cross-polarization [12], and UV-light-generated radicals from the substrate itself, leading to long-lived hyperpolarized solid-state samples [13, 14]. Dedicated transport systems of such samples have been demonstrated for remote dissolution, circumventing the need for an on-site polarizer [15, 16].

### The Rise of Parahydrogen-Based Methods

**Fig. 1:**
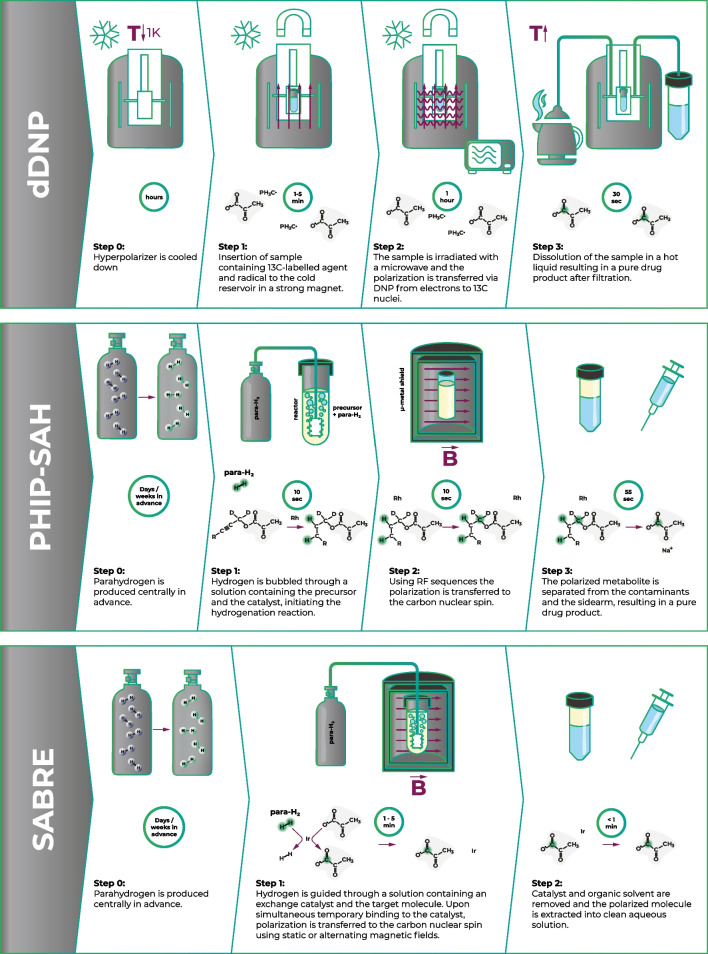
Techniques comparison: d-DNP, PHIP-SAH, SABRE. Comparison between the most established ^13^C polarization methods. d-DNP relies on transfer of polarization from free electrons under conditions of very low temperature and high magnetic field. PHIP-SAH and SABRE utilize parahydrogen as the source of polarization, transferred to the metabolite in a fast chemical reaction

As an emerging option to d-DNP for preclinical and clinical hyperpolarized ^13^C MRI, parahydrogen-based hyperpolarization methods do not require a superconducting magnet and cryogenic temperatures and were discussed with a special focus in our workshop. They potentially offer a more controllable, faster, technically less demanding, and more cost-efficient polarization procedure. Parahydrogen induced polarization (PHIP) was first predicted theoretically in 1986 by Bowers and Weitekamp and demonstrated experimentally the following year [17]. The method relies on parahydrogen as a source of the hyperpolarization, has lower equipment costs compared to d-DNP, and hyperpolarization occurs at a significantly faster rate (Fig. 1) [18–21]. However, the process typically requires organic solvents and the use of a catalyst that must be removed before exposure to the biological system. Therefore, attention has been focused on the attainment of a fully biocompatible aqueous solution of the hyperpolarized metabolite, with recent successful results, although this approach has not yet been approved for human use. Additionally, PHIP has faced limitations due to a small portfolio of agents, which includes succinate, fumarate, and a few others [22–24]. During the workshop, two main strategies based on PHIP were discussed, PHIP-SAH and SABRE, which have expanded the portfolio and are currently being extensively explored.

- Side Arm Hydrogenation (PHIP-SAH)
  This approach, pioneered by Reineri and colleagues [25], allows the application of PHIP to ^13^C-labeled pyruvate and other biologically relevant molecules that contain a carboxylate group (Fig. 1). Feasibility has been demonstrated in metabolic studies in cells and in preclinical *in vivo* studies [26–28].
  Recently published results demonstrated that a PHIP-SAH-based [1-^13^C]pyruvate hyperpolarization method with a novel purification technique achieved ~ 18% polarization at the time of injection, with comparable imaging results to d-DNP in the same animal and with an excellent safety profile over a cohort of 24 mice and 24 rats [29].
- SABRE
  Signal amplification by reversible exchange (SABRE) is a promising parahydrogen-based technique for hyperpolarizing molecules without the need for chemical modification [30] (Fig. 1). While high polarizations have been reported for many compounds [18], SABRE has recently been enabled for hyperpolarization of ^13^C-labeled pyruvate [18, 31–33], achieving up to 22 and 6% polarization of ^13^C at the C1 and C2 positions, respectively (prior to purification) [34]. Additionally, progress in sample purification has yielded clean aqueous pyruvate solutions with > 10% polarization, which have recently been used for the first *in vivo* metabolic MRI experiments in mice [35].
  PHIP-SAH and SABRE both have distinct advantages: SABRE does not require the synthesis of a precursor and thus is expected to be cheaper and to have fewer impurities. However, SABRE currently provides lower polarization and at lower concentrations compared to PHIP-SAH, which presents a challenge in scaling to clinical use. In addition, as the catalyst binding mechanism differs between the two methods, some probes can be more easily polarized with one method or the other. Thus, the two approaches can be considered complementary by providing together a broader range of hyperpolarized probes.

### Preclinical Hyperpolarized ^13^C MR: Current and New Applications

Most of the focus of hyperpolarized ^13^C MR technology to date has been in the field of oncology, both for improved diagnosis and classification of tumors, as well as for monitoring therapeutic responses (see [36, 37] for recent reviews). Preclinical work in cancer is still ongoing to determine the best use cases of the technology. During the workshop, a preclinical study in estrogen receptor positive (ER +) patient-derived breast cancer xenografts treated with a phosphoinositide 3-kinase (PI3K) inhibitor was presented which demonstrated that hyperpolarized [1-^13^C]pyruvate detected response where none could be detected using fluorodeoxyglucose-18 positron emission tomography (^18^F-FDG-PET). The decrease in lactate labeling was shown to be a specific consequence of decreased expression of the transcription factor Forkhead box protein M1 (FOXM1) [38]. Similarly, hyperpolarized [1-^13^C]pyruvate detected early metabolic changes in a murine model of gastric cancer in response to a pan-tyrosine kinase inhibitor when no significant changes were detected by ^18^F-FDG-PET [39].

Beyond oncology, [1-^13^C]pyruvate has been used recently in preclinical studies on neurological pathologies such as neurodegenerative disease and brain trauma. In a mouse model of multiple sclerosis, a new study showed hyperpolarized ^13^C MR imaging of [1-^13^C]pyruvate detected immunological responses to disease-modifying therapies, providing unique information on neuroinflammation and its modulation [40]. Importantly, hyperpolarized ^13^C MRS detected response to dimethyl fumarate therapy, whereas conventional T_1_ post contrast MRI did not, demonstrating the complementarity to conventional MRI. Another recent study showed that specific neuronal deletion of the glucose transporter 3 (GLUT3cKO) or pyruvate kinase 1 (PKM1cKO) in mice hippocampi led to memory impairment and metabolic changes detectable by hyperpolarized ^13^C MRI/S [41]. Female, but not male, PKM1cKO mice had increased hyperpolarized [1-^13^C]pyruvate-to-lactate conversion, while this ratio was decreased in female GLUT3cKO mice. ^18^F-FDG-PET did not detect changes, highlighting the potential for hyperpolarized [1-^13^C]pyruvate to detect downstream alterations in brain glucose metabolism. Expanding on previous work [42–44], another study explored the potential of hyperpolarized ^13^C MR to detect long-lasting alterations in brain metabolism following repetitive mild repetitive traumatic brain injury (rTBI) in mice [45]. Decreased conversion of hyperpolarized [1-^13^C]pyruvate to lactate, linked to decreased pyruvate dehydrogenase activity, was detected in mice after rTBI, which was not detectable with other MRI methods. Machine learning approaches showed that hyperpolarized [1-^13^C]pyruvate can detect long-lasting metabolic impairment resulting from rTBI and predict associated behavioral changes, thereby demonstrating its potential for improving the detection and monitoring of previously undetected rTBI.

In addition to pyruvate, recent work was presented, where alternative hyperpolarized probes were used to explore other metabolic reactions *in vivo* including glutaminolysis, redox status, and fructolysis. Building on previous work [46–48], glutamine metabolism through glutaminase highlights a key way in which hyperpolarized glutamine can provide non-invasive measurement of on-target inhibition of metabolic flux [49]. As hyperpolarized pyruvate is currently used in imaging brain tumors in humans [50], using pyruvate as a solvent for other probes provide a means of overcoming previous limitations. For example, new formulations of hyperpolarized dehydroascorbate using pyruvate as a solvent potentially allow visualization of redox status in the brain [51]. Furthermore, utilizing hyperpolarized fructose [52] as an orthogonal probe of glycolysis can not only highlight differential nutrient utilization by flux through the enzyme ketohexokinase but also flux switching that occurs in cancer [53]. Ultimately, these encouraging results suggest that the field is ready to expand well beyond pyruvate as a probe *in vivo*.

Besides probing metabolism, hyperpolarized ^13^C MR of suitable sensor molecules can be used to probe tissue properties, such as temperature, ion content, redox state, or pH. Even though pH is an important biomarker which can be crucial for disease diagnosis [54] and therapy success [55, 56], there is currently no routinely applied imaging modality in the clinic for measuring tissue pH [57]. Hyperpolarized ^13^C MR has been successfully applied for pH imaging *in vivo* with pH sensors such as bicarbonate [58–60] and zymonic acids [61], which show an intrinsically high sensitivity to pH alterations in the physiological range. Translation of these sensors to the clinic has so far been limited by magnetization lifetime or challenging agent preparation. In contrast to hyperpolarized metabolic agents, hyperpolarized pH sensors only rely on chemical shift differences between pH-sensitive moieties. Acquisition strategies do not have to be dynamic, which enables the design of efficient acquisition techniques for improved spatial resolution. Emerging hyperpolarized pH agents, such as Z-OMPD, will likely unlock the clinical value of pH imaging, for which there is sufficient evidence in a handful of studies [62–64].

### Clinical Studies: Scope, Acquisition, and Analysis

The first paper describing the production of hyperpolarized ^13^C-labeled substrates in solution was published in 2003 [3], and the first clinical patient study with hyperpolarized [1-^13^C]pyruvate 10 years later in 2013 [65]. The research to date has shown that hyperpolarized [1-^13^C]pyruvate has great promise to revolutionize medical diagnostic imaging, and current studies are exploring clinical applications that have significant potential to improve patient outcome.

Most clinical applications have so far been in oncology, where it was shown, that the hyperpolarized [^13^C] lactate signal is higher in aggressive breast, renal, and prostate cancers compared to more benign disease, with potential clinical applicability for patient stratification and for targeting biopsies [5, 66–72]. In gliomas for example, it has been demonstrated that hyperpolarized [1-^13^C]pyruvate can detect metabolic subtypes, which can be dichotomized into more glycolytic and oxidative subtypes that have differing drug and radiation sensitivities [73, 74]. Therefore, imaging glioma patients with hyperpolarized [1-^13^C]pyruvate could be used to help guide treatment selection. Early treatment response assessment is another promising application, where an increase in hyperpolarized [^13^C] lactate labeling after 7–11 days of neoadjuvant chemotherapy has been demonstrated as an early response biomarker in triple-negative breast cancer [75]. Comparison of hyperpolarized imaging to tissue-based metrics of metabolism is revealing the molecular basis for these changes including the role of the pyruvate transporter (MCT1), lactate exporter (MCT4), and the enzyme lactate dehydrogenase (LDH), and can be used to explain intratumoral and intertumoral metabolic heterogeneity on imaging. In prostate cancer, elevated conversion rates of [1-^13^C]pyruvate to [1-^13^C]lactate (k_PL_) have been demonstrated in more aggressive cancers, which can be utilized for improved biopsy guidance for primary organ-confined disease [72]. Another unmet clinical need that hyperpolarized ^13^C MR can potentially address is metabolic imaging of metastatic cancers to lung and bone [76] and detection of response to therapy by assessing lymph nodes and bone metastases in prostate cancer [77]. Exciting new studies have also shown applications of [1-^13^C]pyruvate for clinical research in renal cancer [67]. While most human studies have focused on [1-^13^C]pyruvate, new HP probes have been translated into human studies including: [2-^13^C]pyruvate that can detect metabolism via acetyl-CoA to acetylcarnitine, glutamate, and glutamine [78]; [1-^13^C]alpha-ketoglutarate to detect conversion to glutamate and in mutant IDH cancers to 2-hydroxyglutarate (2-HG) [79]; and [^13^C,^15^N_2_]urea that can be co-polarized with ^13^C-pyruvate to provide unique perfusion and metabolic information simultaneously [80, 81].

Beyond oncology, applications in cardiology are also being investigated. The case for pushing hyperpolarized cardiac magnetic resonance imaging (CMR) is based on the accumulating evidence that the accuracy of conventional myocardial viability assessment is not sufficient [82]. Hyperpolarized [1-^13^C]pyruvate CMR resembles PET/MR exams and as such could potentially replace PET/MR CMR exams in the clinical setting, if the method is either as good as FDG-PET or potentially better due to added metabolic measurements. Hyperpolarized perfusion imaging has the potential to provide as accurate perfusion information as ^15^O-water PET, and it has been demonstrated that rest/stress pyruvate imaging is possible [83]. The current evidence suggests that hyperpolarized CMR holds great potential and that the method is ready for clinical adoption [84–89].

In renal applications, hyperpolarized [1-^13^C]pyruvate exams have the potential to provide accurate metabolic readouts of the underlying metabolic cascades associated with kidney diseases [90]. The chronic nature and slow progression of renal disease and the low sensitivity and specificity of many biomarkers of renal dysfunction limits the ability to treat patients and develop new treatments [91, 92]. The increased sensitivity and specificity that hyperpolarized [1-^13^C]pyruvate MRI provides could potentially be used in clinical trials as earlier markers of disease progression and thus treatment response [93–97]. As anti-fibrotic drugs are entering clinical use, methods to select patients and monitor progression will be key, and hyperpolarized [1-^13^C]pyruvate used in combination with apparent diffusion coefficient (ADC) imaging provides the necessary sensitivity and specificity [98].

Finally, deuterium metabolic imaging (DMI) was discussed during the workshop as a new metabolic imaging technique that can potentially complement hyperpolarized ^13^C MR. DMI was used recently to study both the metabolism of oral [6,6-^2^H_2_]glucose and [2,3-^2^H_2_]fumarate [99–101]. One of the interesting points of discussion raised during the workshop was the interplay between hyperpolarized ^13^C MR and DMI [102], where the community was encouraged to consider what the best application for each method would be and ways in which these methods can complement each other. For example, a direct comparison between [2,3-^2^H_2_]fumarate and hyperpolarized [1,4-^13^C]fumarate on the same animal has not been performed, but comparison of the results of studies [100, 103], utilizing the different agents for assessing response to treatment in the same tumor model and treatment suggests that [2,3-^2^H_2_]fumarate is more sensitive in detecting response to treatment, but also requires a much higher concentration and scan time and achieves a lower malate SNR. As another example, oral [6,6-^2^H_2_]glucose may provide complementary metrics of oxidative and reductive metabolism.

### Considerations on Data Analysis

Complex, dynamic, multidimensional hyperpolarized ^13^C MR is frequently parameterized using pharmacokinetic (PK) models or simpler area-under-the-curve (AUC) metabolite maps, or ratios of AUC maps [7]. Clinical adoption of this technology will require establishment of imaging biomarkers that are robust, reproducible, and readily interpretable. Although AUC maps and AUC ratios are straightforward to calculate, they are affected by a range of physical and physiological factors that may hinder interpretation. PK models facilitate quantification of rate constants that can reduce bias imparted by some of these factors, although care must be taken to select a PK model that balances accuracy and complexity in the context of the application [104]. The effects of confounding factors may also be minimized by careful experimental design and by acquisition of supplemental information to assess the potential impact of key sources of bias [105].

## C. Hyperpolarized ^13^C: where are we going?

**Fig. 2:**
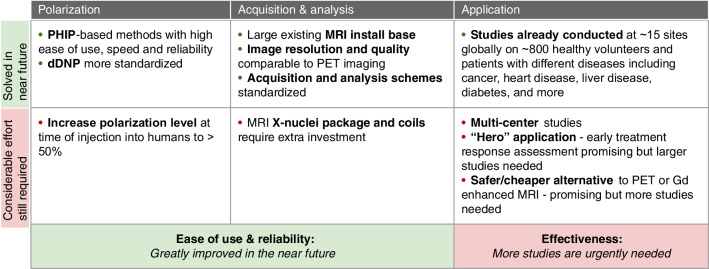
The future of hyperpolarized ^13^C: for widespread use hyperpolarized MRI needs to be easy to use, reliable, and effective. Ease of use and reliability are dependent on technological advances that are already underway and expected to become fully realized in the near future. Evidence of effectiveness requires more and larger clinical studies

The future of hyperpolarized ^13^C technology was at the center of the scientific exchange at the workshop. Key questions that were raised included: Why is not MRI-based metabolic imaging more prevalent today, 10 years after the first publication of d-DNP use in humans? What is needed for widespread use of the technology? What important problems can the technology address? The main discussion points are summarized below and in Fig. 2.

The main point that was agreed upon unanimously was that for the technology to become scalable and impactful, the hyperpolarized ^13^C MR technology has to be easy to use, regardless of the specific methodology and equipment employed. The following aspects were discussed:

- From the perspective of polarization, progress is being made in both d-DNP and PHIP-based polarization techniques, with methods becoming more standardized and streamlined and new polarization technologies becoming newly accessible, opening the way to larger scale preclinical and clinical studies. As such, more evidence is expected to accumulate in the coming years, paving the way towards clinical applications over a wide range of medical fields.
- In addition to polarizers, specific MRI acquisition capabilities are required. Firstly, hyperpolarized ^13^C MR metabolic imaging requires the MRI scanner to have ^13^C transmit and receive capabilities. While almost all small animal MRI systems have X-nuclei capabilities (although this is not provided by the vendors as a default), it is not the case for clinical systems. Among the 35,000 clinical MRI scanners installed world-wide, only approximately 10% have X-nuclei capabilities (cf. https://stats.oecd.org/). Even more importantly, out of these only a few hundred centers are actually utilizing this capacity. This low prevalence and utilization of X-nuclei capability is likely due to the difficulties of implementing X-nuclei MRI or MRS in the clinical setting. For example, for ^31^P MRS, 3-APP was suggested as an exogenous probe for extracellular pH [106, 107]. However, the measurement times for this pH imaging technique were long and the spatial resolution was poor, preventing wide-spread use even though it is still considered a “gold standard” for pH measurements. Furthermore, it is important to note that ~ 70% of the current MRI systems are 1.5 Tesla scanners, and to date, upgrading to X-nuclei capabilities is only available for 3 Tesla systems. This is due to vendor marketing policy and may need to change to enable wider accessibility of hyperpolarized metabolic imaging. Finally, the combined cost of adding X-nuclei capabilities (hardware and software) and radiofrequency coils might be prohibitive for most centers (> $100 K).

After “ease of use,” the group unanimously took the position that robustness and reliability are a major factor required for scalability. As hyperpolarized ^13^C MR becomes easier and more reliable, its use will increase, not only in research but also as a routine clinical tool, in a similar fashion to PET during the 1990s. Given the obvious parallel between PET and hyperpolarized ^13^C MR, a discussion comparing the market access and potential of both methods took place. First, it was pointed out that, despite having been around for 40 years, the impact and accessibility of PET are still modest, with only ~ 5000 PET scanners worldwide, 80% of them in high income countries [108]. The lack of wider dissemination of this technology might partially be due to the need for a cyclotron, dedicated facilities with complex radiation controls, radioactive waste infrastructure and trained staff, which is a prohibitive cost for developing countries. On the other hand, the number of MRI scanners is one order of magnitude higher, with an estimate of 35,000 worldwide, with a number of about 80 million MRI exams performed per year (cf. https://stats.oecd.org/). Like PET, MRI scanners are more prevalent in high income countries, with Japan having the highest ratio of MRI units per million population (57.39 per million in 2020) followed by the USA with 38 units per million in 2021 (cf. https://stats.oecd.org/ and [109]). Finally, an issue often raised in the context of hyperpolarized ^13^C MRI is the achievable spatial resolution, which today is around 6 mm for pyruvate and 12 mm for its products. Although this in-plane resolution is comparable to PET, the out of plane resolution of hyperpolarized ^13^C MRI is still coarse. At the same time, there was agreement that the required resolution is driven by the individual application. As an outlook, denoising approaches are being applied broadly and could help to improve the quality of the images produced [110]. More generally, in the future, machine learning and artificial intelligence may render “image quality” (and thus resolution) less relevant if there will be a shift from relying on radiologists’ interpretation that is based on images to machine-based interpretation of the raw data.

A major part of the discussion focused on potential applications of hyperpolarized ^13^C MR, searching to find the “hero experiments” (a term which the group preferred to “killer applications”). Most participants believed that the biggest potential of this technology was for early treatment response assessment/prediction, especially in oncology. In parallel, a question was raised as to whether it is necessary for the technology to solve a current clinical unmet need to become widely useful or will it suffices to offer a safer and/or cheaper alternative to an existing tool. The recent FDA approval of hyperpolarized xenon for assessment of lung function serves as an interesting test case, as it was approved based on a non-inferiority comparison study to standard radionuclide methods rather than for a new application. One such example for a “non-inferiority” application for hyperpolarized MRI that was discussed is as an alternative contrast agent to gadolinium chelates, especially considering recent growing concerns as to the long-term safety of these agents [111]. Other potential “non-inferiority” applications are those applications for which PET is used today, e.g., in oncology, neurology, and cardiology, as discussed in “Latest Advances in Hyperpolarized 13C MR.”

In conclusion, the participants shared an optimistic view about the future of hyperpolarized ^13^C MR. Recent technological advancement on the polarization side such as the development of PHIP methods, the ongoing efforts to introduce new probes both preclinically and in first-in-man clinical studies, as well as recent community efforts towards standardization of the technology such as the International Society for Magnetic Resonance in Medicine (ISMRM) consensus group initiative and the first attempts at multicenter studies pave the way for hyperpolarized ^13^C MR to become much easier to use and more reliable. Combined with the already widely established and available MRI infrastructure, hyperpolarized MRI has the potential to scale up quickly to more widespread usage. This will hopefully encourage larger scale preclinical and clinical studies that are urgently needed to establish the usefulness of the tool and to explore its full potential.
